# Parkinson-related neuropathy

**DOI:** 10.6061/clinics/2021/e2675

**Published:** 2021-02-01

**Authors:** Josef Finsterer, Fúlvio Alexandre Scorza, Carla Alessandra Scorza, Ana Claudia Fiorini

**Affiliations:** I Klinik Landstrasse, Messerli Institute, Vienna, Austria; II Disciplina de Neurociencia. Universidade Federal de Sao Paulo/Escola Paulista de Medicina (UNIFESP/EPM), Sao Paulo, SP, BR; III Programa de Estudos Pos-Graduacao em Fonoaudiologia, Pontificia Universidade Catolica de Sao Paulo (PUC-SP), Sao Paulo, SP, BR; IV Departamento de Fonoaudiologia, Escola Paulista de Medicina/Universidade Federal de Sao Paulo (EPM/UNIFESP), Sao Paulo, SP, BR

## INTRODUCTION

Although Parkinson’s disease (PD) primarily affects the central nervous system, it is a multi-organ disease which also affects the eyes and the peripheral nervous system, including the autonomic fibers (1,2). PD-related disturbance of the peripheral nerves may result in sensory, motor, or autonomic neuropathy. Autonomic dysfunction can manifest as sicca syndrome, hypo-/hyperhidrosis, orthostatic hypotension, reduced blood pressure variability, reduced heart rate variability, nausea, constipation, vomiting, urinary dysfunction, or erectile dysfunction (1). Non-motor manifestations, such as hyposmia, rapid eye movement sleep behavior disorder (RBD), constipation, or depression, may precede these motor symptoms (1). Although PD-related neuropathy (PDRNP) is well recognized, relatively little data about this topic are available. Thus, this mini-review aimed to summarize and discuss previous and current data to provide an overview of the clinical presentation, diagnosis, frequency, and therapeutic management of PDRNP.

## METHODS

A review of published literature collected by searching the PubMed and Google-scholar databases using appropriate search terms was performed.

## RESULTS

In total, 18 articles dealing with the topic of interest were retrieved (Table 1). Concerning the clinical presentation, patients with PDRNP may complain of motor, sensory, or autonomic symptoms, which can be confirmed by appropriate clinical investigation, autonomic testing, nerve conduction studies (NCSs), and nerve biopsy. In most patients, NCSs revealed an axonal lesion of motor, sensory, or both sensory and motor fibers (Table 1). Hereditary PDRNP is predominantly a large fiber neuropathy, whereas acquired PDRNP manifests frequently as small fiber neuropathy (Table 1). Autonomic testing may reveal cardiovascular autonomic neuropathy or impaired electrochemical skin conductance (3). Thus far, nerve biopsy has not been carried out in patients with PDRNP; therefore, we do not regard nerve biopsy as a cornerstone for diagnosing PDRNP (4) as it is only applied if neuropathy (NP) due to vasculitis, sarcoidosis, amyloidosis, or leprosy is suspected. Although small fiber neuropathy (SFN) can be difficult to diagnose (4), the most sensitive method to detect SFN is skin biopsy (5).

Regarding its etiology, PDRNP is multicausal. It can present due to an underlying genetic defect causing PD and NP, or it may be secondary due to side effects of treatment or concomitant diseases in conjunction with NP (Table 1). Genetic disorders manifesting with PD and NP include mitochondrial disorders (MIDs) (6), spino-cerebellar ataxias (SCAs) (7), and hereditary spastic paraplegia (HSP) (8). An example of an MID manifesting with PDRNP is multisystem MID due to mutations in *POLG1* (Table 1) (6). Various mutations in *POLG1* that manifest phenotypically with PDRNP have been found (9). In addition to *POLG1* variants, mitochondrial PDRNP may also be due to mutations in *C10orf12* (*twinkle*), *MPV17*, and *SLC25A46,* or in mtDNA related genes (Table 1). An example of a HSP manifesting with PDRNP is HSP39 due to mutations in *PNPLA6* (8). An example of SCA manifesting with PDRNP is SCA48 due to mutations in *STUB1* (7).

PDRNP may also be caused by long-term usage of L-DOPA. L-DOPA may not only cause vitamin-B12 deficiency (L-DOPA induced vitamin-B12 deficiency), but also folate deficiency (10). The notion that L-DOPA causes vitamin-B12 or folate deficiency, and thus secondary PDRNP, has been challenged by findings from third world countries showing that low vitamin-B12 and folate levels do not play a significant role in the development of PDRNP (11). It has been increasingly recognized that levidopa/carbidopa intestinal gel (LCIG) can be complicated by NP, particularly SFN (12,13). In a study of 33 patients treated with LCIG, three patients developed symptomatic PDRNP and seven developed subclinical PDRNP (13).

Diagnosis of NP relies on the history, clinical exam, blood tests, NCSs, electromyography (EMG), and autonomic testing (4). EMG may serve as a supplementary method to explore the effects of motor neuropathy on the skeletal muscles.

## CONCLUSIONS

The etiologic spectrum of PDRNP is wider than anticipated, and genetic causes need to be increasingly considered. Diabetes or anti-Parkinson medications should not be readily considered as the most frequent cause of PDRNP to avoid overlooking genetic causes, and a thorough genetic work-up should be implemented.

## Figures and Tables

**Table 1:** Causes of NP in patients with PD.

Etiology	Fiber size	Fiber types	NCSs	Reference
Acquired				
L-DOPA	LFN	sensory	axonal	(14)
LCIG	SFN	sensory	normal	(12)
Duodopa	LFN	sensory-motor	axonal	(15)
Diabetes	SFN/LFN	sensory-motor	axonal	(16)
Thyroid disease	SFN/LFN	sensory-motor	axonal	(16)
B12-deficiency	SFN/LFN	sensory	axonal	(16)
Hereditary				
PARK2	LFN	sensory, HNPP	axonal	(17)
FMR1	LFN	sensori-motor	axonal	(18)
ATXN3	LFN	sensori-motor	axonal	(19)
ATP13A2	LFN	nr	axonal	(20)
STUB1	LFN	nr	nr	(7)
FIG4	LFN	sensori-motor	demyelinating	(21)
SNCA	SFN	sensory	normal	(22)
GBA	LFN	motor	axonal	(6)
POLG1	LFN	motor	axonal	(6)
C10orf12	LFN	sensory-motor	axonal	(23)
MPV17	LFN	sensori-motor	axonal	(24)
SLC25A46	LFN	sensori-motor	nr	(25)
12S-rRNA	LFN	sensory	axonal	(26)
tRNA(Lys)	LFN	sensory	axonal	(27)
ND4	LFN	nr	axonal	(28)

DOPA: Dihydroxyphenylalanin, LCIG: levodopa/carbidopa, LFN: large fiber neuropathy, NCSs: nerve conduction studies, nr: not reported, SFN: small fiber neuropathy, NP: neuropathy, PD: Parkinson’s disease.
